# Improved Local Search with Momentum for Bayesian Networks Structure Learning

**DOI:** 10.3390/e23060750

**Published:** 2021-06-15

**Authors:** Xiaohan Liu, Xiaoguang Gao, Zidong Wang, Xinxin Ru

**Affiliations:** School of Electronics and Information, Northwestern Polytechnical University, Xi’an 710129, China; lxhwwey@mail.nwpu.edu.cn (X.L.); nwpu_wzd@mail.nwpu.edu.cn (Z.W.); ru@mail.nwpu.edu.cn (X.R.)

**Keywords:** probabilistic graphical models, structure learning, local search

## Abstract

Bayesian Networks structure learning (BNSL) is a troublesome problem that aims to search for an optimal structure. An exact search tends to sacrifice a significant amount of time and memory to promote accuracy, while the local search can tackle complex networks with thousands of variables but commonly gets stuck in a local optimum. In this paper, two novel and practical operators and a derived operator are proposed to perturb structures and maintain the acyclicity. Then, we design a framework, incorporating an influential perturbation factor integrated by three proposed operators, to escape current local optimal and improve the dilemma that outcomes trap in local optimal. The experimental results illustrate that our algorithm can output competitive results compared with the state-of-the-art constraint-based method in most cases. Meanwhile, our algorithm reaches an equivalent or better solution found by the state-of-the-art exact search and hybrid methods.

## 1. Introduction

A crucial matter in artificial intelligence is the development of models learned from data that can provide a structural representation based on the domain knowledge and have their own definite semantics. Undoubtedly, Bayesian networks (BNs) [1] have been a practical and versatile tool for addressing this issue. For example, they have been extensively used for probabilistic inference in medical decision support [2,3], speech recognition [4], adaptive robot control [5], gene expression in bioinformatics [6], and so forth.

BN uses a graphed-based representation as the basis for compactly encoding a complex distribution over a high-dimensional space. Concretely, it is composed of a directed acyclic graph (DAG) where nodes correspond to the variables, and the directed edges correspond to direct probabilistic interactions between them. Therefore, the BN learning problem consists of the DAG recovery and numerical parameters characterizing it, called BN structure learning (BNSL) and BN parameter learning (BNPL), respectively. The former is considered more challenging than the latter because searching for an accurate graphical representation is NP-hard [7]. Existing BNSL methods can be categorized into three types: constraint-based, score-based, and hybrid approaches. Firstly, constraint-based methods, such as PC [8,9], use a series of conditional hypothesis tests to learn independence. Hence, the approaches involve numerous samples and are restricted by the hypothesis test adopted. On the other hand, score-based methods identify a DAG by optimizing a score function, typically based on the log-likelihood of statistics. Besides, there are also hybrid approaches, which first prune the search space using a constraint-based method, followed by a greedy search for an optimal structure. Max-min hill-climbing (MMHC) [10] is one of the most well-known of these methods. Unfortunately, hybrid methods are not as fantastic as the score-based algorithm in the optimization aspect, nor as fast as the constraint-based algorithm [11]. Thus, this paper focuses on the score-based methods. The score-based approaches further operate in two different search methods. The first approach tends to obtain an optimal solution for BNSL, called exact search. Several exact algorithms have been developed based on dynamic programming [12,13,14], branch and bound [15], linear and integer programming [16,17,18], shortest-path heuristic [19,20,21] in the past decades. However, the exact search is hindered by a burdensome task, the complete exploration of the space of possible parent sets, whose time and memory usages are growing exponentially. When the number of variables exceeds 50, the mainstream algorithms become unpractical, or the algorithm’s parameters need to be changed, which will sacrifice some possibility of searching higher scores. The second one, called local search, is generally based on heuristics over the DAG space, such as hill-climbing (HC) [22]. Furthermore, local search has been carried out over the space of equivalence classes of the network structures [23,24] and the space of topological sorts (or the ordering space) [25,26]. A drawback of these algorithms is generally trapped in local optimum solutions, thereby offering an approximate solution to BNSL. Intuitively, enhancing the performance of the greedy local search by Iterated Local Search (ILS) [27] can improve this dilemma. Lee and van Beek have used ILS to obtain a fancy improvement over ordering space [28]. However, the ordering space is a simplified expression of the DAG space in dimension, which restricts the search space and loses some probabilities of finding a better solution [29].

In this paper, we apply ILS over the DAG space itself rather than any alternatives of DAG. In general, it is thought to be tough acting on the structure by complex operators due to acyclicity. Thus, two novel operators, the Leaf operator and the Root operator are proposed to make it possible to perturb the structure itself directly and maintain the acyclic constraint. Moreover, we propose a derivative operator, Swap operator, to form Momentum factor with the Leaf and Root operators. In contrast to existing primary perturbation factors, the Momentum factor we propose can affect multiple edges in one operation, which traverses a more extensive search space and is more conducive to escaping the local optimum. Then we propose a framework that incorporates the Momentum factor and ILS to eliminate the limitation of the local optimum partially. As shown by our experiment results, our algorithm is capable of finding networks that score significantly better than other state-of-the-art solvers in given instances.

The paper is organized as follows: Section 2 covers the background of BN and some concepts of BNSL. We introduce our method, the operators proposed and their properties in Section 3. Then Section 4 presents and discusses the results. Finally, Section 5 provides our concluding remarks and directions for future research.

## 2. Background

### 2.1. Bayesian Networks

We start with some background on Bayesian networks. A Bayesian network is a probability graph model, which can be expressed as BN{G,P}, where *P* is the probability distribution of nodes, representing the intensity of causality between nodes. *G* is a DAG whose nodes represent random variables $X_{1},X_{2},\ldots ,X_{n}$. In a DAG *G*, if there is a directed edge $X_{i}\to X_{j}$, then $X_{i}$ is identified as a parent of $X_{j}$ and $X_{j}$ is a child of $X_{i}$. Nodes without children are called leaf nodes, and nodes without parents are named root nodes. Let ${Pa}_{X_{i}}^{G}$ denote the parents of $X_{i}$ in *G*, and ${Nondescendants}_{X_{i}}$ denote the variables in the graph that are not descendant of $X_{i}$. Then *G* encodes the following set of conditional independence assumptions, called the local independencies, and denoted by $I_{l}\left(G\right)$: for each variable $X_{i}$: $\left(X_{i}⊥{Nondescendants}_{X_{i}}|{Pa}_{X_{i}}^{G}\right)$. In other words, the local independencies state that each node $X_{i}$ is conditionally independent of its nondescendants given its parents [30]. Formally, BN can be considered as a recipe for factorizing a joint distribution of $\left\{X_{1},X_{2},\ldots ,X_{n}\right\}$:

(1) $$P\left(X_{1},X_{2},\ldots ,X_{n}\right)=\prod_{i=1}^{n}P\left(X_{i}|{Pa}_{X_{i}}^{G}\right).$$

Additionally, to facilitate further understanding, some common concepts are given:

**Definition** **1.** *Markov equivalence: Two network structures, G and G’, are Markov equivalent if the set of distributions represented by one of the DAGs is identical to the set of distributions that can be represented by the other.*

The Markov equivalence definition designates that two equivalents *G* and *G’* are statistically indistinguishable in BNs.

**Definition** **2.** *V-structure: Three variables $X_{1}$, $X_{2}$, and $X_{3}$ form a v-structure if $X_{2}$ has two incoming edges from $X_{1}$ and $X_{3}$, forming $X_{1}\to X_{2}\leftarrow X_{3}$ while $X_{1}$ is not adjacent to $X_{3}$.*

Two equivalents, *G* and *G’*, share a set of v-structures as the same skeleton and partial structure.

**Definition** **3.** *Maximum in-degree: The maximum number of parents allowed for a variable, called maximum in-degree.*

This is a local property in the graph, usually constrained for managing the exploration complexity in BNSL problem.

**Definition** **4.** *Maximum out-degree: The maximum number of children allowed for a variable, called maximum out-degree.*

In contrast to the maximum in-degree, the maximum out-degree is not used as a constraint. It is suggested here to facilitate discussion on operator effect, proposed in the next section.

### 2.2. Scoring Function

The most used approach, score-based, is to find the best DAG according to some scoring functions, which are available to measure the degree of fitness between BN structure and the dependencies of variables. Broadly, the Bayesian Dirichlet equivalent uniform (BDeu) [31], minimum description criterion (MDL) [32], Bayesian information criterion (BIC) [33], Akaike Information Criterion (AIC) [34] are most adopted in BNSL. Except for MDL (the lower MDL is, the better BN structure is), when other scoring functions are used, learning the optimal structure can be expressed as:

(2) $$G^{*}=\underset{G}{\mathrm{argmax}}\;\;score\left(G,D\right),$$

where *D* is the given dataset. All of the scoring functions previously mentioned share the critical property of decomposability: The score of a DAG is constituted by the sum of the scores of the subgraphs made by each variable $X_{i}$ with its parents ${Pa}_{X_{i}}^{G}$:

(3) $$score\left(G,D\right)\;=\;\sum_{X_{i}}score(X_{i},Pa_{X_{i}}^{G},D).$$

Profiting the property, a local change in the structure (such as adding, deleting, or reversing an edge) does not change the score of other parts of the structure that remained the same.

In this paper, we adopt BIC to evaluate the posterior probability of the candidate structures:

(4) $$\mathrm{BIC}\left(G\right)\;=\;\sum_{i=1}^{n}\sum_{\pi \in \left|{\mathrm{Pa}}_{X_{i}}^{G}\right|}N_{x,\pi }\log{\hat{\theta }}_{x|\pi }-\frac{\log N}{2}\left(\left|X_{i}\right|-1\right)\left(\left|{\mathrm{Pa}}_{X_{i}}^{G}\right|\right),$$

where *n* implies the number of variables and *N* is the instances number of a complete data set. ${\hat{\theta }}_{x|\pi }$ is the maximum likelihood estimate of the conditional probability $P(X_{i}=x|{Pa}_{X_{i}}^{G}=\pi )$, and π denote the values of $X_{i}$ and ${Pa}_{X_{i}}^{G}$, respectively. $N_{x,\pi }$ represents the number of times $\left(X=x\wedge {Pa}_{X_{i}}^{G}=\pi \right)$ appears in the dataset and $\left|\cdot \right|$ indicates the number of states of variables and $\left|⌀\right|\;=\;1$.

## 3. Methodology

### 3.1. ILS over the DAG Space

As a greedy search over the DAG space, the hill-climbing algorithm is typically stuck in local optimal or cannot cross the plateau. Fortunately, ILS has historically performed competitively with other metaheuristic methods by a simple and intuitive extension of basic hill-climbing [27]. Compared with the random restart HC method, the ILS considers searching for an improvement close to the local optimum rather than a simple random restart. More tangibly, a random DAG is chosen as an initial candidate solution, and subsequently, a local optimum is found based on chosen DAG through local search. It is the first step and found preparations for consequent iteration. Then the algorithm iterates in three steps until the termination condition is fulfilled: first, the current local maximum is perturbed through the perturbation factor. Second, a new local optimal is established based on the perturb solution through local search. Finally, comparing the two solutions found before, the best one is chosen as the new one. The algorithm stops when a given termination condition is fulfilled.

Adopting ILS over DAG space to obtain a structure *G*, we construct the ILSG algorithm, and the pseudocode of ILSG is provided in Algorithm 1. Regarding the perturbation factor of ILSG, $p_{f}$, it is the number of operators used to perturb *G*. In detail, we sample an operator from the Add operator, Delete operator, Reverse operator to impact on *G*, which is carried out $p_{f}$ times in each iteration. These operators represent adding an edge for *G*, deleting an edge for *G*, and reversing an edge for *G*, respectively. For legality of the result, if the operators break the acyclic constraint, remove the operation or operate it in the opposite way. For example, if the Add operator breaks the acyclic constraint, the edge added by Add operator will be reversed. To avoid getting stuck in sluggishness, ILSG will restart from a new random structure if the soft restart condition is satisfied. A soft restart emerges when the current solution has not achieved the new one over $s_{r}$ iterations.
**Algorithm 1** ILSG algorithm**input:** Dataset *D* **Output:** Optimal structure $G^{*}$ and its score BIC $\left(G^{*}\right)$ 1: G←randomDAG(D) 2: G←localsearch(G) 3: $G^{*}\leftarrow emptygraph\left(D\right)$ 4: **while** (termination condition is not met) **do** 5:   $G^{\prime }\leftarrow perturb\left(G\right)$ 6:   $G^{\prime }\leftarrow localsearch\left(G^{\prime }\right)$ 7:   $G\leftarrow compare(G,G^{\prime })$ 8:   $G^{*}\leftarrow compare\left(G,G^{*}\right)$ 9:   **if** soft restart condition is met **then** 10:     G←randomDAG(D) 11:   **end if** 12: **end while** 13: **return**
$G^{*}$, BIC $\left(G^{*}\right)$


### 3.2. More Complex Operators

The causal role of the perturbation factor in eliminating the restriction of the local optimum has been demonstrated above, but the contribution of primary perturbation factors is inadequate. A high-level intuition is that if a compound perturbation, like the momentum, applies to the current solution, more opportunities to reach a better solution will arise. Unfortunately, the acyclic constraint makes it impossible for complex operators to be applied to DAG due to the fact that seeking a cycle in a DAG involves exponential time.

**Figure 1:**
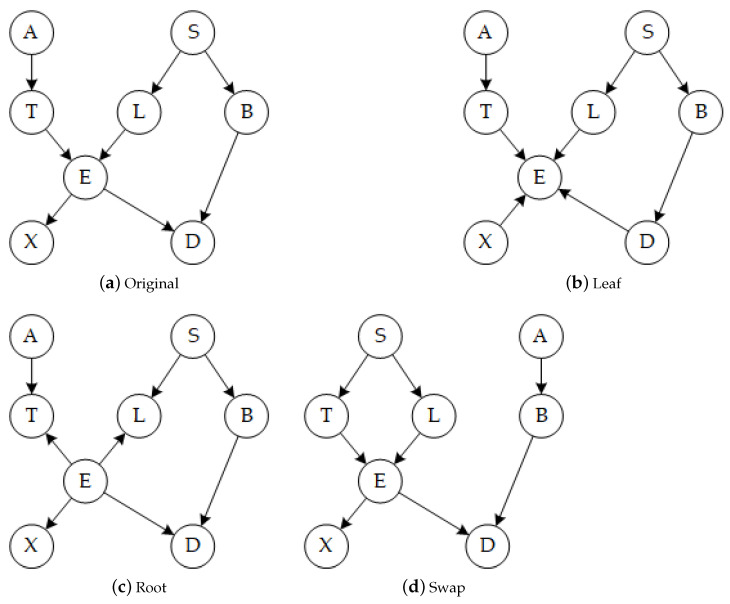
The representation of operators: (**a**) The original DAG; (**b**) The DAG after an action: Leaf (*E*); (**c**) The DAG after an action: Root (*E*); (**d**) The DAG after an action: Swap (*T*, *B*).

In order to facilitate the operation of DAG, we regard the edges as a matrix composed of two lists: *from-list* and *to-list*. Individually, we view an edge as consisting of a *from-node* and a *to-node*. Then we propose three operators, the Leaf operator, the Root operator and the Swap operator to complicate DAG. Besides, revealing the effect of the operators to be introduced more intuitively, all operators are applied to a classic benchmark network Asia, and the results are shown in Figure 1. Among them, Figure 1a shows the original structure of the Asia network.

#### 3.2.1. Leaf Operator

**Definition** **5.** *Leaf (X) operator: converting the node X into a leaf node.*

It is not challenging to implement the Leaf operator, which needs three steps:

1. All leaf nodes of DAG are found to avoid invalid operations.
2. A node *X* to be processed is sampled without replacement from the remaining nodes.
3. If the *from-node* of an edge is the selected node *X*, the edge is reversed by the Reverse operator.

Figure 1b shows the consequence of the Leaf operator that two edges are reversed, and node E is converted into a leaf node.

**Theorem** **1.** *The Leaf operator satisfies acyclic constraint.*

**Proof** **of Theorem 1.** Suppose that the operation, Leaf (*X*), violates the acyclic constraint. Namely, it generates a cycle in the DAG *G*. Then there must be a path: X→…→X in *G*, which indicates *X* must be one of the parents of a node. However, it is contradictory that *X* has been a leaf node.    □

**Corollary** **1.** *When other operators break the acyclic constraint, the cycle can be eliminated by acting the Leaf operator on the from-node of the edge generating the cycle.*

**Proof** **of Corollary 1.** It is obviously true, since the *from-node* of the edge generating the cycle, has been transferred as a leaf node, while the leaf node does not change the acyclicity according to Theorem 1.    □

If we consider the effect of basic operators: Add, Delete, Reverse on structure as one unit, a fundamental property of the Leaf operator is:

**Property** **1.** *Denote the maximum out-degree of a DAG as u. The interval of the effect on the structure is (1, u).*

**Proof** **of Property 1.** Consider two extremes, the worst and the best. The worst condition is that the node operated by the Leaf operator appears only once in the from-list, which affects the structure by 1 unit. By contrast, the best one is that the node operated by the Leaf are root nodes with the maximum out-degree *u*, which affects the structure in *u* units.    □

#### 3.2.2. Root Operator

**Definition** **6.** *Root (X) operator: converting the node X into a root node.*

The difference in the implementation process between Leaf and Root is that the operation object of Root changes to the *to-node*. Figure 1c shows the effect of the root operator that two edges are reversed, and node E is transformed to a root node. It is similar to Leaf but Root has the following key feature:

**Theorem** **2.** *The Root operator satisfies acyclic constraint.*

**Proof** **of Theorem 2.** Suppose that the operation, Root (*X*), breaks the acyclic constraint, namely, generates a cycle in DAG. Then there must be a path: X→…→X. In other words, *X* must be one of the children of a node. It is contradictory that *X* has been a root node.    □

Then the complexity discussion of the Root operator is provided:

**Property** **2.** *Denote the maximum in-degree of a DAG as k. The interval of the effect on the structure is (1, k).*

**Proof** **of Property 2.** Still consider two extremes, the worst and the best. The worst condition is that all nodes operated by the Root operator appear only once in the *to-list*, which affects the structure by 1 unit. On the other hand, the best one is that the nodes operated by the Root are leaf nodes with the maximum in-degree *k*, which affects the structure in *k* units.    □

Likewise, the Root operator can also eliminate cycles generated by other operators. However, the maximum out-degree is commonly larger than the maximum in-degree. In other words, eliminating cycles through the Leaf can explore more extensive space than the Root. Consequently, we tend to satisfy acyclicity by the Leaf instead of Root.

#### 3.2.3. Swap Operator

**Definition** **7.** *Swap (X, Y) operator: swapping two nodes, X and Y, in the to-list.*

The Swap operator is considerably different in complexity from the above operators. The algorithm implementing the Swap operator is provided in three stages:

1. The black and white lists are ascertained according to node *X*.
2. The node *Y* to be swapped is sampled from the white list.
3. To guarantee the legality of the result, resample the node *Y* if it breaks the legitimacy of DAG.

**Figure 2:**
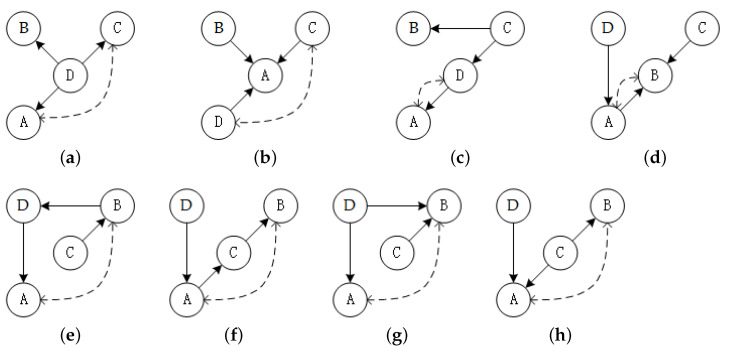
Representations of all meaningless and illegal operations, and the dash line indicates two nodes to be swapped. (**a**) meaningless swap: Swap (*A*, *C*); (**b**) meaningless swap: Swap (*C*, *D*); (**c**) illegal swap: Swap (*A*, *D*); (**d**) illegal swap: Swap (*A*, *B*); (**e**) illegal swap: Swap (*A*, *B*); (**f**) illegal swap: Swap (*A*, *B*); (**g**) illegal swap: Swap (*A*, *B*); (**h**) illegal swap: Swap (*A*, *B*).

Figure 1d shows the influence of the Swap operator. It is different from the first two operators because the Swap operator may break acyclic constraint. Fortunately, the cycle can be eliminated by addressing the node which generates the cycle through Leaf, according to Corollary 1. All meaningless and illegal swap operations are shown in Figure 2, which constructs the foundation of the blacklist of the first step and the legitimacy test of the third step. In detail, Figure 2a,b are meaningless swaps because the exchanged nodes share the same child node or parent node; Figure 2c,d are illegal swaps, which leads to the illogical case that the parent node and the child node are the same; Figure 2e,f represent a prohibited condition that generates bidirectional edges; Figure 2g,h show a banned situation that generates existing edges. Then the complexity discussion of the Swap operator is provided:

**Property** **3.** Denote the maximum out-degree of a DAG as u. The interval of the effect on structure is (4, 4+2*u).

**Proof** **of Property 2.** Still consider two extremes, the worst and the best. The worst condition is that the pair of nodes operated by the Swap does not break the acyclic constraint, which affects the structure by 4 units. In contrast, the best one is that two nodes operated by the Swap break acyclic constraint, and every Leaf operator used to eliminate the cycle has the best impact, which affects the structure by 4+2*u units.    □

**Figure 3:**
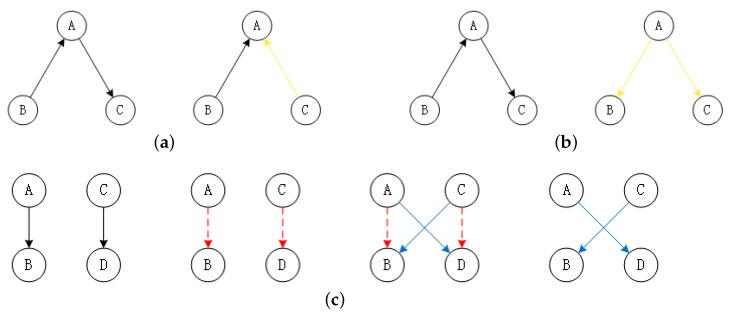
Representations of the relationship between the three operators proposed above and three basic operators. Yellow lines denote Reverse operations; red dash lines indicate Add operations; blue lines mean Delete operations. (**a**) Leaf; (**b**) Root; (**c**) Swap.

In fact, as shown in Figure 3, the proposed three operators can be equivalently regarded as a combination of a series of basic operations. Specifically, the Leaf and Root can be considered to compose some corresponding Reverse operations as shown in Figure 3a and Figure 3b. Likewise, as shown in Figure 3c, the Swap operator can be viewed as consisting of two Delete operations, two Add operations, and some possible reverse operations (when the acyclic constraint is broken). Although the three operators proposed above are more complex than fundamental operations, the effect may be weakened by multiple actions on the same DAG. As a simple example, there is an edge A→B in a DAG. First, we convert node *A* into a leaf node by Leaf (*A*). Then we use the Leaf operator for the DAG again. Unfortunately, node *B* is selected, which leads to all the operations in vain for edge A→B. Therefore, we design a factor, integrating the operators to affect the structure more powerfully and overcome the drawback.

### 3.3. ILSG with Momentum

On the basis of the more complicated operators, Leaf, Root, Swap, we propose a compound factor, Momentum, to motivate the algorithm ILSG to reach a better solution from the local optimum. The implementation of the Momentum is provided in Algorithm 2. Furthermore, the framework is suggested by combining Momentum with ILSG (ILSM) and improving some details, and the pseudocode of ILSM is sketched in Algorithm 3.
**Algorithm 2** Momentum algorithm**input:** A DAG *G* to be processed, the number *m* of objects operated by Momentum
**Output:** The DAG *G’* has been processed 1: $S_{node}\leftarrow getnodes\left(G\right)$ 2: **for**
i∈(1,m)
**do** 3:    $X_{i}\leftarrow sample\left(S_{node}\right)$ 4:    operator←sample(Leaf,Root,Swap) 5:    $G^{\prime }\leftarrow op\left(X_{i}\right)$ 6: **end for** 7: **return**
*G’*


The algorithm begins with a local optimum generated through ILSG. Then, until the termination condition is met, ILSM obtain a new solution by ILSG over and over. When the number of variables increases, a given number of perturbation factors of ILSG becomes impractical. Hence the parameter, a trade-off between practical and efficiency, is designed to step with the algorithm ILSM gradually. The stride of perturbation factors is designed to be an array related to the number of instances. When the stepping condition is satisfied, the stride will be stepped according to the value in the array. Such as, stride∈{n/10,n/5,n/2,n}, objects influenced by perturbation factors of ILSG are *n*/10 at the beginning of ILSM. It will step to *n*/5 to obtain a mightier perturbation if the step condition is satisfied. ILSM is operated by Momentum to avoid stagnation and restarted from a new initial graph according to an operation and restart schedule. The Momentum acts on the structure if the objective value has not been improved over $r_{s}$ moving to a new local optimum. After $r_{h}$ Momentum operations, the restart condition is fulfilled, and ILSM restarts from a new random graph.
**Algorithm 3** ILSM algorithm**input:** Dataset *D*, the number *m* of objects operated by Momentum
**Output:** Optimal structure $G^{*}$ and its score BIC ($G^{*}$) 1: G←randomDAG(D) 2: G←ILSG(G) 3: $G^{*}\leftarrow emptygraph\left(D\right)$ 4: **while** termination condition is not met **do** 5:   $G^{\prime }\leftarrow ILSG\left(G,stride\right)$ 6:    $G\leftarrow compare(G,G^{\prime })$ 7:    $G^{*}\leftarrow compare\left(G,G^{*}\right)$ 8:    **if** the condition of changing stride is met **then** 9:       stride++ 10:    **end if** 11:    **if** the condition of using Momentum is met **then** 12:      G←Momentum(G,m) 13:    **end if** 14:    **if** the restart condition is met **then** 15:      G←randomDAG(D) 16:    **end if** 17: **end while** 18: **return**
$G^{*}$, BIC ($G^{*}$


## 4. Experimental Evaluation

In this section, we first introduce the score criteria, other algorithms, and cases that will be experimented for comparison. Then, we perform experiments to show the effect of three powerful operators, Leaf, Root, Swap, and the compound factor Momentum combined by them, compared to the primary operators, Add, Delete, Reverse. Later, we compare our method ILSM to other existing BNSL algorithms on eight classical BNs. The operators proposed by us and ILSM were implemented in R language, and the experiments were run on a computer with Windows 10, an Intel Core i5-8300H (2.30 GHz) processor with four cores, eight threads, and 8GB of RAM.

### 4.1. Scoring Metrics

BIC and two structural metrics are considered to evaluate the accuracy of the learned graph. The introduction of BIC has been provided in Section 2.2. The two structural metrics use varying combinations of the following parameters [35]:

- True Positives (TP): the number of edges in the learned graph also present in the true graph.
- True Negatives (TN): the number of direct independencies discovered in the learned graph exist in the true graph.
- False Positives (FP): the number of edges in the learned graph not present in the true graph.
- False Negatives (FN): the number of edges not in the learned graph but present in the true graph.

The first structural metric, called the Structural Hamming Distance (SHD) [10], compares the structure of the learned and the original networks. It represents the number of steps required to transform the learned graph into the original graph, namely,

(5) SHD=missingedges+extraedges+incorrectlyorientededges

where a score of 0 indicates a perfect fitting between the learned and the true graph.

The second metric, called the Balanced Scoring Function (BSF) [36], is a recent metric that considers all four parameters and returns a fully balanced score. Formally,

(6) $$\mathrm{BSF}\;=\;0.5\times (\frac{TP}{a}+\frac{TN}{i}-\frac{FP}{i}-\frac{FN}{a}),$$

where *a* is the number of edges, and *i* is the number of direct independences in the actual graph:

(7) $$i\;=\;\frac{\left|V\right|\left(\left|V\right|-1\right)}{2}-a,$$

where $\left|V\right|$ is the size of the variable set V. The score ranges from −1 to 1, where a score of −1 corresponds to the least accurate graph, a score of 1 to a graph that is a perfect match of the ground-truth graph, and 0 to an empty or a fully connected baseline graph.

### 4.2. Benchmark Data Sets of Experiments

In order to embody the performance of algorithms in networks with the different number of variables, we choose two small networks (<20 variables), two medium networks (20–50 variables), two large networks (50–100 variables), very large networks (>100 variables). The sources and the judgement criteria of these benchmarks’ scale are from the BN repository [35]. They are all well-known benchmark data sets, and Table 1 summarizes the characteristics of the true networks for them.

### 4.3. BNSL Algorithms Considered

In Section 4.4.2, the performance of ILSM is assessed with reference to seven algorithms that have been applied to the same data. The algorithms selected represent state-of-the-art in the overview [37] or well-established implementation. Specifically,

1. PC-stable [9]: a modern and stable implementation of the state-of-the-art constraint-based algorithm called PC.
2. IAMB-FDR [38]: a variant of IAMB, a constraint-based algorithm based on discovering Markov Blanket, adjusts the tests significance threshold with FDR. In the following it is abbreviated as IAMB.
3. HC: the most popular local search algorithm adopted over the DAG space. As the results of HC are consistently unstable and not enough to compare with other solvers, we choose HC with restart but still abbreviate it as HC.
4. MMHC: perhaps the most popular hybrid learning algorithm that combines the Max-Min Parents and Children algorithm and HC.
5. Hybrid HPC (H2PC) [39]: a hybrid algorithm combines the HPC (to restrict the search space) and the HC (to find the optimal network structure in the restricted space).
6. SaiyanH [40,41]: a recent novel and state-of-the-art hybrid algorithm combines a constraint-based phase with an associational score Mean/Max/MeanMax marginal Discrepancy and HC. In the following it is abbreviated as Saiyan.
7. GOBNILP: it is a current state-of-the-art exact search approach based on integer linear programming. In the following it is abbreviated as ILP.

The bnlearn R package version 4.6.1 (https://www.bnlearn.com, accessed on 15 June 2021) was used to test algorithms 1 to 5. The SaiyanH algorithm was tested using the Bayesys open-source BNSL system version 2.42 (http://bayesian-ai.eecs.qmul.ac.uk/bayesys, accessed on 15 June 2021). Finally, ILP was tested using the GOBNILP software version 1.6.3 (https://www.cs.york.ac.uk/aig/sw/gobnilp, accessed on 15 June 2021).

### 4.4. Experimental Results and Discussion

#### 4.4.1. Comparisons of Operators

As the structures of Asia and Sachs were elementary, we compared the proposed operators with three basic operators on the virtual networks of the other six benchmark datasets. Although BSF was more balanced than SHD, the latter corresponded to the proposed quantitative estimation of the effects of operators in Section 3.2. Consequently, we chose SHD as the criterion to estimate the impact between operators in this section. Moreover, the purpose of this section was to find which operator or factor had the most powerful influence on the structure instead of comparing accuracy, thus we thirsted for a higher SHD.

Every operator experimented with 5*n times in each true network for guaranteeing to get as many states as possible, where n was the number of nodes. The maximum, minimum, and mean values of SHD are shown in Table 2. No matter how many calculations were made, the influence of the three basic operators (Add, Delete, Reverse) on the structure was 1.00, so their results are listed in one column in Table 2.

Obviously, the influence interval of Leaf and Root on the structure was consistent with that derived in Section 3.2, and the result of Swap did not reach the extreme situation of theoretical derivation in Section 3.2. Nevertheless, it was still within the range of theoretical derivation. Further, Swap’s mean effect was usually more potent than that of Leaf and Root, which was due to its min SHD of 4.00. However, the structure became more and more complex with the increase of variables, and the max effect of Swap was poorer than that of Leaf. Such as, in Win95pts, Pathfinder, Andes networks (the three most complex networks in our experiments) Swap’s max values of SHD were worse than that of Leaf. It was not complicated to understand in theoretical aspects because the more complex the structure was, the more laborious it was to violate the acyclic constraint by a simple swap. Each of the three operators had its advantages, but they could not be dominant in all aspects, which was one of the reasons why we aspired to propose the integrated factor: Momentum.

Testing the performance of the Momentum, we carried out four groups of comparisons, and the number of objects affected by the operators in each group was *n*/5, *n*/2, *n*, 2*n, respectively. Namely, $\mathrm{operator}{\left(round(m)\right)}_{m=n/5,n/2,n,2*n}$, where m was the number of objects and *n* was the number of variables, *round()* indicated rounding off all of non-integer. For instance, Add(*n*/5) denoted adding *n*/5 edges for structures, Leaf(*n*/2) indicated converting *n*/2 nodes into leaf nodes, Swap(*n*) signified exchanging n pairs of nodes, Momentum(2*n) meant operating 2*n object through Momentum. The comparison results are shown in Table 3.

As in Table 3, the integrated factor, Momentum, naturally had the most significant effect on the structure of almost all benchmarks. A closer look at the experimental data revealed that Momentum played a dominant role in affecting the structure when the number of objects was few, such as *m* = *n*/5, *n*/2, though it was tied with Swap for *m* = *n*, 2*n. As analyzed in Section 3.2, the influence of Leaf and Root were hardly more potent with increasing *m* in benchmarks with an uncomplicated structure. For example, Leaf(*n*/5) even was poorer than basic operators in the Hailfinder network. Moreover, the effect of Leaf(*n*/2), Leaf(*n*), Leaf(2*n) were nearly equal in the Insurance network. Therefore, by alternating the three proposed operators, we could eliminate the limitation of a single operator’s drawback, which was also one of the reasons we desired to propose Momentum. It shall be noticed that the results of Swap were competitive, which indicated the performance of Swap did not significantly weaken with increasing m. Nevertheless, there was a gap between Swap and Momentum, which was reflected in the average distance between the results of Momentum and Swap, which was 32.5. As shown in Table 3, though Momentum indeed improved the performance of operators with multi-objects, it was dispensable to increase objects to 2*n. Therefore, the subsequent experiments adopted Momentum(*n*) rather than Momentum(2*n), a trade-off between time complexity and actual effect.

**Figure 4:**
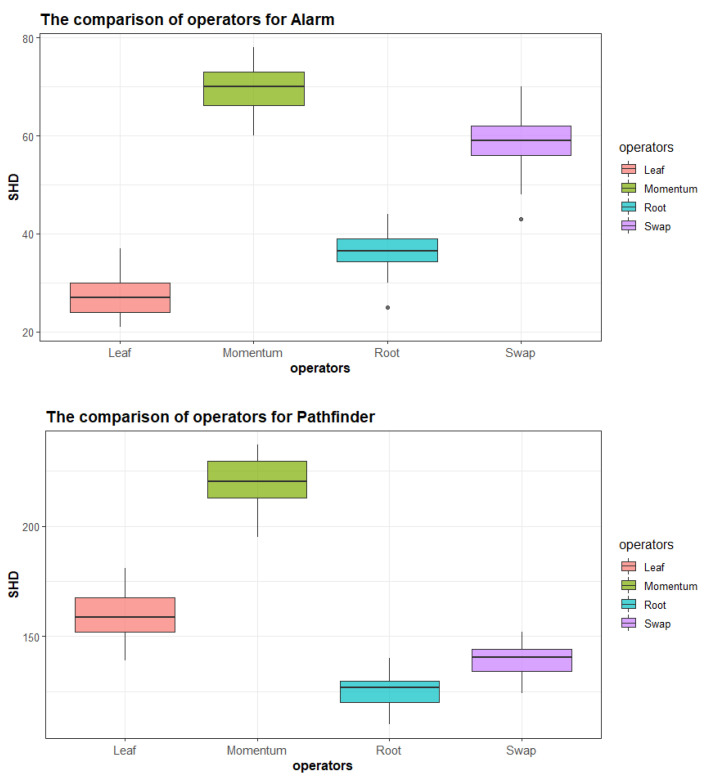
The comparisons of operators for two benchmarks. The time of experiments: 50.

Furthermore, comparing Momentum’s robustness and exhibiting Momentum’s superiority more intuitively, we performed many groups on Momentum and three proposed operators with *n* objects based on the classical Alarm and Pathfinder, the representative of the complex and medium complex network. Figure 4 shows the performance of three proposed operators and Momentum.

Intuitively, Momentum was dominant in both less complex and complex networks. In detail, the median SHD metric based on Momentum was about 17.9% and 39.7% higher than that of the suboptimal operator, and about 71.9% and 155.5% higher than that the most inefficient operator for two benchmarks, respectively. Moreover, for the Alarm network, some extreme values existed in the Swap and Root results, whereas Momentum’s results were very compact. Overall, Momentum could steadily affect the structure in a potent way.

#### 4.4.2. Comparisons of Algorithms

We varied the size of dataset N∈{1000,10,000,50,000,100,000} in the benchmark networks mentioned in Section 4.3. Besides, we set the maximum in-degree to *k* = 6, a high value that allows learning even complex structures. It shall be noticed that our approach did not involve a maximum in-degree. Evaluating the best performance of algorithms, we ran each solver for 6 hours in all following experiments. The parameters of ILSM were tuned and their optimal values are listed in Table 4.

**Figure 5:**
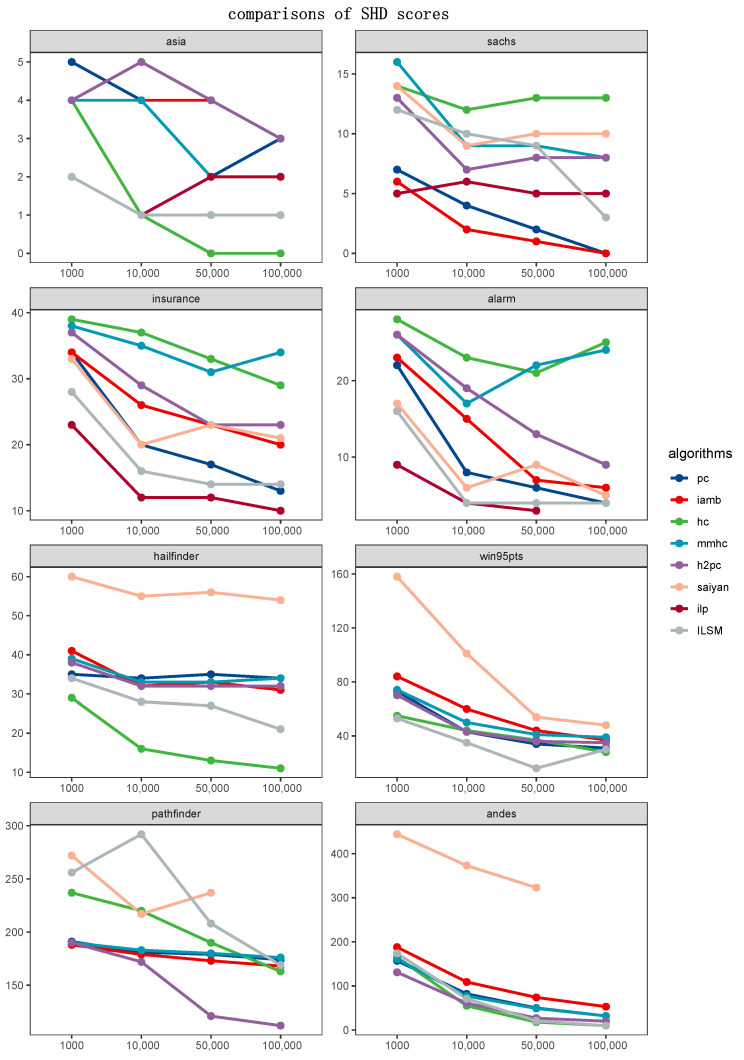
Comparisons of SHD scores.The x-axis of each graph denotes sample sizes of the input data, whereas the y-axis represents SHD scores (lower is better).

**Figure 6:**
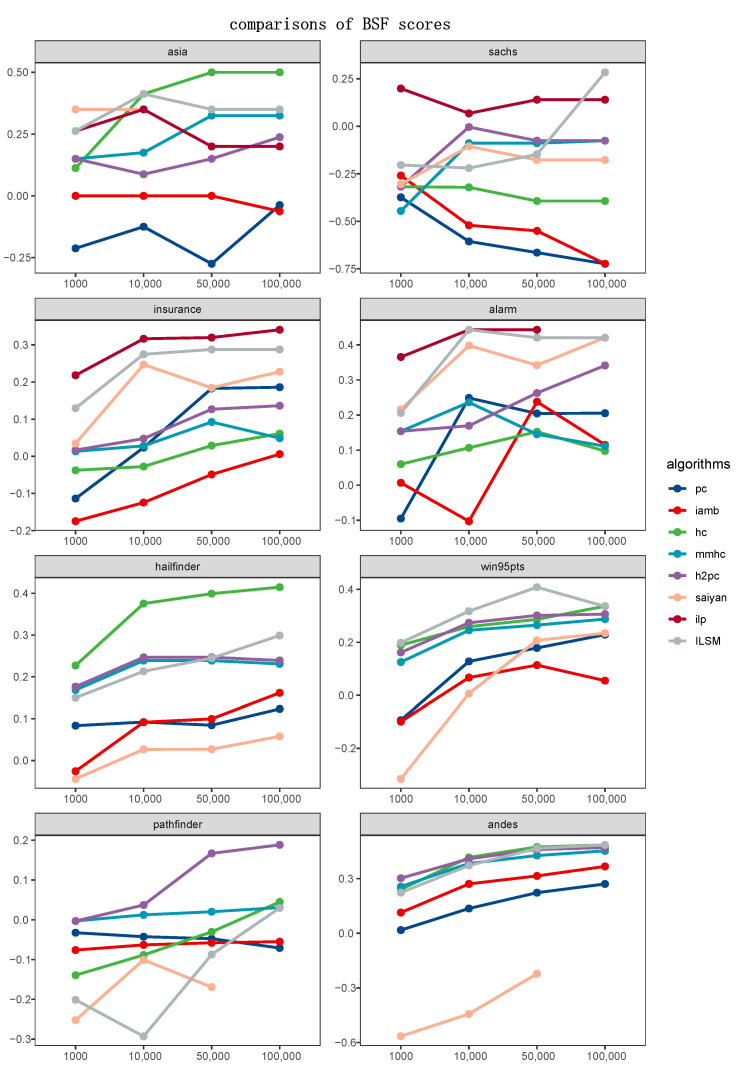
Comparisons of BSF scores.The x-axis of each graph denotes sample sizes of the input data, whereas the y-axis represents BSF scores (higher is better).

To qualitatively analyze which algorithm could learn a DAG closer to the ground-truth structure, we compared ILSM and algorithms mentioned in Section 4.3. Figure 5 and Figure 6 displayed the BSF and SHD metric of ILSM with reference to the scores produced by the other seven algorithms, respectively. Each of the 16 graphs corresponded to a case study and a metric. Unlike the experiments in Section 4.4.1, which measured the effect of the operators, a lower SHD score showed a better performance in this section. It was important to note that, in contrast to SHD, a higher BSF score demonstrated better performance. Some lines were incomplete or missing, which illustrated that the corresponding algorithm failed to learn a structure in the time limitation or memory limitation.

A rather interesting outcome in Figure 5 and Figure 6 is that ILP could not handle the n>50 networks with given parameters. Except for ILP, PC, IAMB and ILSM outperformed other methods in most cases. In detail, PC carried out conditional independent tests on the variables, then identified the v-structure and equivalent classes to learn an optimal BN structure, which focused on exploring the distribution of data with respect to the causal relationships between the variables. Similarly, IAMB pursued to detect the Markov Blanket. Thus, it was a matter of course to learn a result with the lower SHD scores for constrained-based algorithms. Unfortunately, it only indicated that the skeleton of their results got closer to that of target networks rather than the results themselves. As a result, PC and IAMB significantly scored worse outcomes in comparing BSF scores in Figure 6. On the contrary, ILP (in processable conditions) and ILSM performed well regardless of comparing SHD or BSF scores. As a score-and-search method, ILSM indeed obtained a somewhat worse evaluation occasionally, such as the Sachs case and Pathfinder case. That was because the network with the highest score did not always match the real network, and it was hard to guarantee the ground-truth network entirely fall into the global optimum solution with the given training data. Further, an actual path did not always acquire a high score, although the training data were generated from the standard network. As a greedy local search method, HC was more unstable despite combining the restart, which was shown as it performed best in 2/8 cases and worst in 3/8 cases. Concerning hybrid methods, their performance was distinctly worse than ILSM, ILP, and two constrained-based algorithms, as shown in Figure 5 and Figure 6.

Aiming to weigh the performance of optimization, namely, which algorithm could achieve the optimal solution or closer to the optimal solution, Table 5 provides an overview of comparing the BIC scores of learned networks by eight networks. It was noted that PC and IAMB might return a Partial Directed Acyclic Graph, which contained undirected edges. However, only when the dependence relations were definitely direct the decomposable score could be calculated.

The most striking result to emerge from Table 5 was that ILSM achieved the highest scores in all 32 cases. More concretely, as an exact solver, ILP could find a global optimum when the maximum in-degree k was set as the default value. However, to guarantee ILP could get a result, we set *k* to the default value in Asia and Sachs cases and *k* = 6 for other networks, otherwise ILP could only tackle the two networks in our experimental conditions. Therefore, as it could be seen from Table 5, ILP achieved the optimal global solution in Asia and Sachs benchmarks but reached a local optimal lower than results of ILSM in Insurance and Alarm cases, and failed to obtain an outcome in Hailfinder, Win95pts, Pathfinder and Andes cases. Regarding hybrid approaches, it was similar to comparisons of SHD and BSF where the results of MMHC and H2PC were not as outstanding as score-based methods. In detail, the score gap between the results of ILSM and MMHC was 53.7%, and it was 35.0% for ILSM and H2PC, whereas it was 1.9% for ILP. Although Saiyan performed worse in comparisons of SHD and BSF, it commonly could find a competitive structure in small, medium, and large networks, which was revealed in Table 5 as a bit of gap between Saiyan’s results and ILSM’s results, whereas the gap sharply increased to 18.9% and 27.6% for Pathfinder and Andes benchmarks. Saiyan solver could not output a learned network even if it ran out of the time limitation in Pathfinder100000 and Andes100000 cases. Incorporating the Momentum factor, ILSM distinctly improved the condition that local search ordinarily got stuck in local optimal by comparing ILSM and HC with the restart. It was presented in Table 5 as ILSM found better structures than HC for 28/32 instances. It was noticed that the structures found by ILSM and HC were equivalent or tied in Asia and Andes cases, which indicated the impact produced by Momentum was redundant in the simplest network, while it was inadequate in the networks with a sheer number of variables. Fortunately, such networks generally were rare.

## 5. Conclusions

We propose two new operators, Leaf and Root, and a derivative operator, Swap, which can influence the DAG itself rather than any alternatives or simplified expressions of structures. Then, a new framework, ILSM, based on ILS, is proposed that incorporates the Momentum factor integrated by three proposed operators. Experiments have demonstrated that the ILSM can guide a better solution than state-of-the-art hybrid methods and a local search solver with the restart, which indicates our method indeed eliminates the limitation of local optimal to a certain extent. ILSM also reaches a globally optimal solution found by the state-of-the-art exact solver ILP in terms of the small networks, and outperforms ILP in any medium-scale and large-scale instances.

ILSM shares a critical problem with other algorithms based on ILS, that is, they involve many iterations to escape local optimal, which is undoubtedly inefficient. Thus, eliminating the limitation of local optimum in a more heuristic way is a vital issue to tackle.

Our further works include how to expand the impact on the structure to escape the local score maximum and search for a metaheuristics method to promote the efficiency of our method. A memetic search method may be an appropriate choice, but adopting it in the DAG space will be a thorny topic we need to tackle in the future.

## Figures and Tables

**Table 1:** The characteristics of BN benchmarks data sets *.

Benchmark Data Sets *D*	Nodes *n*	Edges *e*	Parameters θ	Max In-Degree *k*	Max Out-Degree *u*
Asia	8	8	18	2	2
Sachs	11	17	178	3	6
Insurance	27	52	984	3	6
Alarm	37	46	509	4	5
Hailfinder	56	66	2656	4	16
Win95pts	76	112	574	7	10
Pathfinder	109	195	77155	5	106
Andes	223	338	1175	6	12

* The true networks of all eight data sets are known, and they are publicly available (http://www.bnlearn.com/bnrepository, accessed on 15 June 2021).

**Table 2:** The impact of operators. Add/Delete/Reverse is abbreviated as A/D/R. The results had been quoted in two decimal places. Unit: 1 SHD.

Network	A/D/R	Leaf			Root			Swap		
	Max/Min/Mean	Max	Min	Mean	Max	Min	Mean	Max	Min	Mean
Insurance	1.00	7.00	1.00	2.36	3.00	1.00	2.10	11.00	4.00	4.69
Alarm	1.00	5.00	1.00	1.86	4.00	1.00	1.84	7.00	4.00	4.30
Hailfinder	1.00	16.00	1.00	1.74	4.00	1.00	1.68	20.00	4.00	4.40
Win95pts	1.00	10.00	1.00	1.74	7.00	1.00	2.66	5.00	4.00	4.00
Pathfinder	1.00	106.00	1.00	6.15	5.00	1.00	1.81	15.00	4.00	4.06
Andes	1.00	12.00	1.00	1.76	6.00	1.00	2.39	9.00	4.00	4.54

**Table 3:** The comparison of operators for the given number of objects. The column labels represent operators, and bold indicate the effect on the structure was the most powerful. Unlike Table 2, three basic operators are listed in Table 3 for comparison. Units: 1 SHD.

*m*	Network	Add	Delete	Reverse	Leaf	Root	Swap	Momentum
*n*/5	Insurance	5	5	5	6	8	14	**27**
	Alarm	7	7	7	9	11	29	**42**
	Hailfinder	11	11	11	9	18	30	**58**
	Win95pts	15	15	15	14	32	52	**86**
	Pathfinder	22	22	22	41	44	**60**	58
	Andes	45	45	45	61	100	164	**252**
*n*/2	Insurance	14	14	14	25	24	45	**57**
	Alarm	19	19	19	15	24	42	**66**
	Hailfinder	28	28	28	32	32	64	**89**
	Win95pts	38	38	38	44	77	97	**149**
	Pathfinder	55	55	55	70	87	84	**192**
	Andes	112	112	112	116	201	340	**422**
*n*	Insurance	27	27	27	28	38	61	**72**
	Alarm	37	37	37	28	36	70	**78**
	Hailfinder	56	56	56	32	50	104	**113**
	Win95pts	76	76	76	63	88	164	**178**
	Pathfinder	109	109	109	175	140	140	**216**
	Andes	223	223	223	165	263	500	**541**
2*n	Insurance	54	52	52	29	47	73	**83**
	Alarm	74	46	46	30	38	89	**90**
	Hailfinder	112	66	66	44	61	110	**125**
	Win95pts	152	112	112	66	108	191	**204**
	Pathfinder	218	195	195	179	174	157	**253**
	Andes	446	338	338	199	317	613	**632**

**Table 4:** Parameters of ILSM.

Parameter	Description	Value
stride	The stride array of perturbation factors of ILSG	{*n*/10, *n*/5, *n*/2, *n*}
$m_{o}$	Objects operated by the factor, Momentum	*n*
$r_{s}$	Number of non-improving steps until acting by Momentum	20
$r_{h}$	Number of Momentum factors until a restart	5

**Table 5:** Comparisons of the capability of each algorithm to search for the optimal solution. “–” means that the score of the corresponding result could not be calculated. Besides, “OM” indicates the solver runs out of memory before any solution was output. “OT” representes that the solver could not output a solution within the time limitation. Bold denotes the score that was the best found amongst all methods. Metric: BIC score (higher is better).

Instances	PC	IAMB	MMHC	H2PC	Saiyan	HC	ILP	ILSM
Asia1000	–	−2410.4	−2369.3	−2369.3	−2203.5	−2212.4	**−2200.6**	**−2200.6**
Asia10000	–	−24,678.5	−24,011.0	−24,240.1	−22,394.2	**−22,392.1**	**−22,392.1**	**−22,392.1**
Asia50000	–	−122,774.1	−120,324.2	−121,476.3	−111,397.4	**−111,397.4**	**−111,397.4**	**−111,397.4**
Asia100000	–	–	−242,298.2	−242,345.3	−223,830.3	**−223,830.3**	**−223,830.3**	**−223,830.3**
Sachs1000	–	–	−8048.8	−7787.6	−7680.0	−7690.7	**−7668.8**	**−7668.8**
Sachs10000	–	–	−74,294.1	**−72,665.5**	−72,678.6	−72,825.0	**−72,665.5**	**−72,665.5**
Sachs50000	–	–	−363,763.4	**−359,445.9**	−359,459.9	−359,633.0	**−359,445.9**	**−359,445.9**
Sachs100000	–	–	**−718,719.1**	**−718,719.1**	−18,737.0	−718,915.0	**−718,719.1**	**−718,719.1**
Insur1000	–	–	−15,613.8	−15,370.5	−14,476.1	−14,485.4	−14,630.2	**−14,370.7**
Insur10000	–	–	−145,705.6	−137,720.0	−135,150.0	−134,404.2	−133,976.6	**−133,644.2**
Insur50000	–	–	−715,325.3	−662,998.8	−667,893.0	−658529.1	−657,485.5	**−657,234.2**
Insur100000	–	–	−1,460,299.6	−1,326,283.6	−1,332,294.9	−1,314,347.5	−1,311,824	**−1,311,582.2**
Alarm1000	–	–	−14,210.2	−14,307.3	−11,675.8	−11,760.8	−11,800.73	**−11,576.3**
Alarm10000	–	–	−125,264.2	−127,175.8	−106,235.6	−107,115.9	−106,262.6	**−106,194.5**
Alarm50000	–	–	−636,475.3	−529,251.3	−525,259.2	−526,975.0	−525,047.2	**−525,033.2**
Alarm100000	–	–	−1,343,931.0	−1,051,838.8	−1,046,071.1	−1,048,645.6	OM	**−1,045,778.0**
Hail1000	–	–	−58,949.9	−59,485.9	−53,739.2	−53,140.4	OM	**−53,129.9**
Hail10000	–	–	−574,164.7	−579,448.2	−505,657.5	−498,475.5	OM	**−498,175.8**
Hail50000	–	–	−2,864,980.1	−2,889,387.7	−2,513,498.6	−2,466,687.4	OM	**−2,466,261.8**
Hail100000	–	–	−5,695,300.5	−5,775,235.6	−5,015,818.9	−4,923,431.1	OM	**−4,923,074.2**
Win1000	–	–	−12,789.4	−13,135.8	−10,935.8	−10,089.8	OM	**−10,009.5**
Win10000	–	–	−114,141.8	−110,574.7	−94,886.9	−92,044.7	OM	**−91,505.9**
Win50000	–	–	−538,549.6	−530,922.4	−472,611.6	−454,162.3	OM	**−453,026.7**
Win100000	–	–	−1,086,467.3	−1,066,673.8	−939,455.3	−902,280.4	OM	**−902,066.0**
Path1000	–	–	−54,335.0	−52,546.5	−43,024.6	−35,421.7	OM	**−34,899.7**
Path10000	–	–	−561,876.7	−431,256.3	−305,996.3	−285,837.6	OM	**−280,420.0**
Path50000	–	–	−2,757,244.8	−1,798,515.5	−1,412,298.9	−1,287,241.1	OM	**−1,276,740.2**
Path100000	–	–	−5,348,864.0	−3,237,761.9	OT	−2,504,395.6	OM	**−2,482,500.9**
Andes1000	–	–	−100,713.3	−98,971.9	−131,976.9	−95,568.9	OM	**−95,560.6**
Andes10000	–	–	−958,957.8	−954,721.2	−1,013,158.6	−933,735.1	OM	**−933,719.5**
Andes50000	–	–	−4,817,602.8	−4,738,844.0	−4,823,995.3	−4,645,111.4	OM	**−4,645,108.9**
Andes100000	–	–	−9,500,828.1	−9,429,374.3	OT	**−9,291,994.9**	OM	**−9,291,994.9**

## Data Availability

The true networks of all eight data sets are known, and they are publicly available (http://www.bnlearn.com/bnrepository, accessed on 15 June 2021).

## Abbreviations

The following abbreviations are used in this manuscript:

BIC	Bayesian information criterion
BNPL	Bayesian Networks parameter learning
BNs	Bayesian Networks
BNSL	Bayesian Networks structure learning
BSF	Balanced Scoring Function
DAG	directed acyclic graph
FN	False Negatives
FP	False Positives
GOBNILP	global optimal Bayesian Networks Integer linear programming
HC	Hill-climbing
ILS	Iterated local search
ILSG	ILS adopted over DAG
ILSM	ILSG with Momentum
MMHC	Max-Min Hill-climbing algorithm
TN	True Negatives
TP	True Positives
SHD	Structural Hamming Distance
